# The Small and the Dead: A Review of Ancient DNA Studies Analysing Micromammal Species

**DOI:** 10.3390/genes8110312

**Published:** 2017-11-08

**Authors:** Roseina Woods, Melissa M. Marr, Selina Brace, Ian Barnes

**Affiliations:** Department of Earth Sciences, Natural History Museum, Cromwell Road, London SW7 5BD, UK; Rosie.Woods@nhm.ac.uk (R.W.); Melissa.Marr@nhm.ac.uk (M.M.M.)

**Keywords:** ancient DNA, small mammals, micromammals, palaeogenetics

## Abstract

The field of ancient DNA (aDNA) has recently been in a state of exponential growth, largely driven by the uptake of Next Generation Sequencing (NGS) techniques. Much of this work has focused on the mammalian megafauna and ancient humans, with comparatively less studies looking at micromammal fauna, despite the potential of these species in testing evolutionary, environmental and taxonomic theories. Several factors make micromammal fauna ideally suited for aDNA extraction and sequencing. Micromammal subfossil assemblages often include the large number of individuals appropriate for population level analyses, and, furthermore, the assemblages are frequently found in cave sites where the constant temperature and sheltered environment provide favourable conditions for DNA preservation. This review looks at studies that include the use of aDNA in molecular analysis of micromammal fauna, in order to examine the wide array of questions that can be answered in the study of small mammals using new palaeogenetic techniques. This study highlights the bias in current aDNA studies and assesses the future use of aDNA as a tool for the study of micromammal fauna.

## 1. Introduction

Techniques to enable extraction and sequencing of DNA from museum specimens, zooarchaeological and sub fossil material [1] have continued to develop since the initial successful recovery of ancient DNA (aDNA) in 1984 [2]. Whilst many of the earliest aDNA studies focused on taxonomy and resolving the phylogenetic placement of extinct species, the development of new techniques, and particularly the use of next generation sequencing (NGS), has allowed the field to expand into ancient pathogens [3,4], population demographic studies [5], conservation issues [6], domestication events [7] and palaeoecological studies [8]. The field of aDNA has, however, largely focused on mammal species [9,10], with a strong bias towards large, charismatic species such as mammoths [11,12], and humans and their closest relatives [13,14,15]. This bias, however, does little to reflect real world species diversity with its plethora of small-bodied mammals. For example, half of all placental diversity is contained within the order Rodentia, a highly speciose order principally composed of small bodied taxa [16].

Here, we define small-bodied or micromammals as any mammal species under 500 g. Definitions of micromammals have typically included members of several orders including Rodentia, Eulipotyphla and occasionally Lagomorpha, Carnivora (family: Mustelidae), and Chiroptera [17]. There is a wealth of research questions that can be addressed via the study of micromammal fauna. These species show rapid generational turnover [18], which is associated with a high mutation rate [19,20,21] and an associated ability to swiftly adapt to new and/or changing environments [22]. There is a strong correlation between mutation rate and systematic components of mammalian life history traits, with rates varying consistently with parameters associated with body size, population dynamics and lifestyle [23]. Mitochondrial substitution rates within the Rodentia, for example, have been estimated to be around 20 times faster than those observed in the Cetacea (whales, dolphins and porpoises), the latter of which show larger body mass, increased longevity and slower generational turnover times [21]. This allows the identification of population level, biogeographic and adaptive responses to past changes in both biotic and abiotic environmental variables. Small bodied mammals typically have high dispersal abilities [24]—a factor that facilitates frequent island colonisation events and thus further facilitates studies relating to biogeography and speciation. Micromammals are also considered key biostratigraphic indicators, providing a source of valuable proxy data with which to infer palaeoecological, palaeoclimatic and palaeogeographical change [25,26,27]. This is due to their widespread abundance in faunal assemblages, their excellent fossil record, and their rapid rate of morphological and genetic change [28,29].

Whilst micromammals can make excellent model study species covering both diverse areas of interest and important concepts in evolutionary biology, palaeontology and conservation, they have been vastly under-represented in ancient DNA studies. Consequently, a review to highlight the potential of working with aDNA from small mammals is long overdue. This review catalogues past efforts to use aDNA to study micromammals and discusses prospects for the future of this field.

## 2. Methods

**Figure 1:**
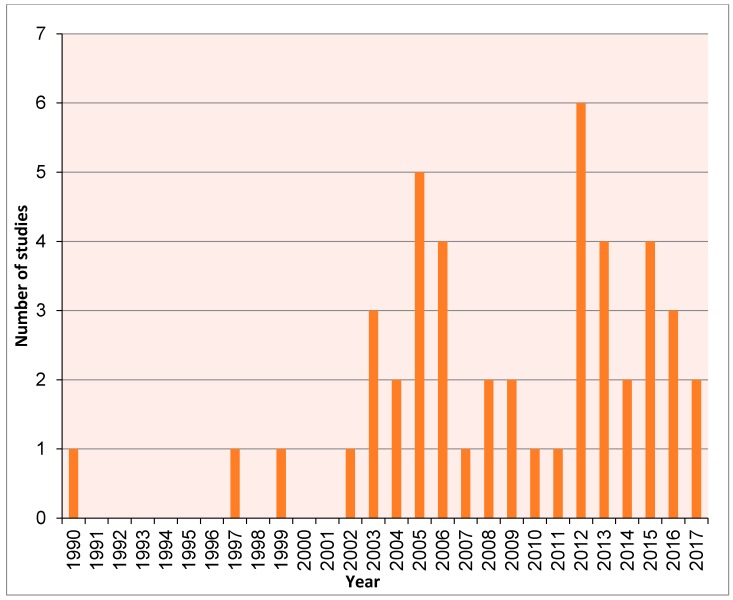
Number of ancient DNA (aDNA) studies including micromammals from 1990–2017.

**Figure 2:**
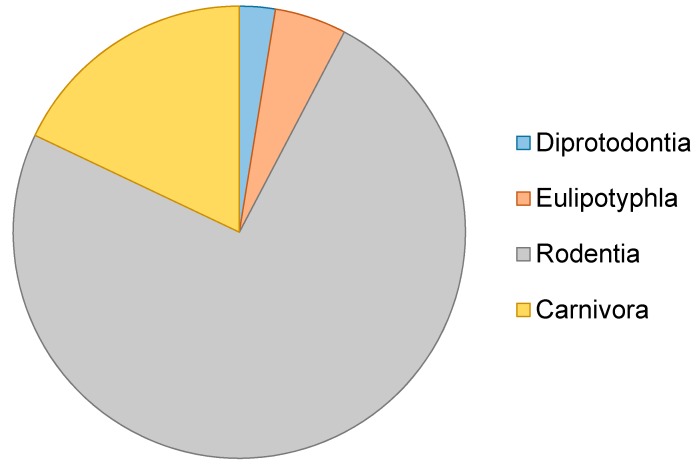
aDNA studies including micromammals between 1990–2015.

This review includes a meta-analysis literature review of studies that utilise ancient DNA techniques to examine micromammal fauna, encompassing a range of materials from degraded museum specimens to sub-fossils. Articles compiled for this review were extracted from ISI Web of Science, PubMed and Google Scholar in 2017 using several search terms including: “ancient DNA”, “aDNA”, “historic DNA”, “palaeogenomic”, “paleogenomic”, “palaeogenetic”, “paleogenetic”, “museum” and “micromammal”, “micro mammal”, “small mammal”. We then assessed a number of articles over time (Figure 1) and potential taxonomic bias (Figure 2).

## 3. Results and Discussion

The following sections of this review address key trends associated with ancient DNA studies utilising micromammal fauna. The list is not exhaustive but rather represents the major themes observed in relation to: technology, source material and the varied questions that small mammal aDNA studies are being employed to address.

### 3.1. Next Generation Sequencing

The study of ancient DNA has been revolutionised by the emergence of new technologies, namely the shift from PCR and traditional Sanger sequencing to NGS [30]. This shift has impacted the field in numerous ways including the challenges faced by researchers hoping to extract endogenous genetic material from degraded specimens. Previous PCR based studies relied on designing primers, a difficult task when studying extinct species with no previously recorded molecular data. The benefit of species-specific primers however is that they target both the region and species of interest, thus reducing the likelihood of sequencing contaminant DNA. In contrast, NGS shotgun approaches have no specificity, with all DNA in a sample, endogenous and contaminant being sequenced. As an alternative to shotgun sequencing, target capture enrichment techniques can be applied prior to sequencing to increase the specificity of the sequencing process, but, as with PCR, the process requires reference sequences from which to design the “baits” to capture the DNA [31,32]. Different micromammal species have different levels of potential for these approaches. For species that are widely used in genetic studies (such as rats or mice), reference sequences are available, for other less studied, rare or extinct species, reference sequences may be unavailable, restricting analyses to either de novo assembly or aligning to the evolutionarily closest (presently often quite distant) sequenced relative [33,34].

### 3.2. DNA Metabarcoding

Micromammal remains, particularly rodents and insectivores, are often found in large quantities that provide limited morphological value. These large assemblages are perhaps optimally analysed using metabarcoding to retrieve viable data for ecological, taxonomic or biogeographical studies. Metabarcoding permits mass sequencing of genetic data using NGS. This technique uses universal PCR primers to mass-amplify DNA barcodes from large collections of specimens or from environmental DNA (eDNA). The resulting sequence data can then be verified and identified to species or genus level using a database of existing DNA data. A study by Guimaraes et al. [35] successfully used DNA barcoding to identify rodent species from a variety of differently preserved specimens and as part of this study correctly identified 75.9% of museum samples and 81.8% of species from owl pellets. The study additionally analysed up to 44,000-year-old rodent material from a North African cave, illustrating that barcoding can also be applied to samples typically identified as difficult to sequence.

### 3.3. Cave Sites

Micromammal remains are often found in cave environments where they form a large proportion of recovered faunal elements [36]. This may be the result of several factors including ecology (the cave was the natural habitat of the species), taphonomy (the small mammals were prey species for some cave-living avian or mammalian predator), or demography (the larger population size of small mammals simply increases the likelihood of their preservation). DNA recovery also seems to be higher in material from cave sites, possibly due to the low variance in temperature which may help to slow down hydrolytic processes involved in DNA degradation, thus preserving DNA that may, outside of the cave environment, have become further fragmented [37,38]. Many aDNA studies including Guimaraes et al. [35], have taken advantage of cave site remains. Gutiérrez-García et al. [39] is a further study where aDNA was successfully analysed from rodents (in this study *Ototylomys phyllotis*) that were excavated from a cave site in a hot/humid climate. This further highlights that micromammals are an excellent study species for aDNA studies, particularly in places such as the tropics where DNA preservation outside of cave environments is poor.

### 3.4. Avian Pellets

Recent and palaeontological pellets from raptors, particularly owls, can provide a valuable source of micromammal skeletal material. Recent pellets can provide a non-invasive method to sample current populations [40], whilst palaeontological pellets can provide a temporal snapshot of the micromammal fauna from a specific place and time [17]. Skeletal and dental material recovered from owl pellets can show excellent morphological preservation in some instances [41,42,43]. However, many studies require identification to species level and this can be problematic unless taxonomically informative elements, which are also in a good state of structural preservation, are recovered. In the absence of such specimens (i.e., in cases where only uninformative postcranial bones are recovered from the pellet), molecular methods may be the most feasible means to address ecological, biogeographical and taxonomic questions.

Pellets are commonly used to find and collect genetic material for extant micromammal populations, as they can provide large numbers of individuals and a variety of species. They can additionally be employed to estimate the geographic distribution of micromammals if the range of the predator species is known. An initial study by Taberlet and Fumagalli [44] successfully sequenced micromammal DNA from owl pellets, but found that the DNA quality was poor. A second study that applied specific aDNA techniques further suggested that extracting DNA from even the most recent specimens recovered from owl pellets warranted an aDNA approach [45]. A recent study [46] confirmed that the quantity of DNA preserved in owl pellets was low but additionally reported high variability in preservation between bones even within the same pellet. It therefore seems likely that the continued development of aDNA techniques will serve to increase the use of owl pellets as a valuable source of genetic data for the study of micromammal fauna, but that the inclusion of a screening step may prove useful to account for the variability in DNA preservation.

### 3.5. Resolving the Phylogenetic Placement of Extinct Species

aDNA studies have been particularly useful in resolving the phylogenetic placement of rare or extinct groups or individual species. In the case of specious groups, such as insectivores and rodents, aDNA has helped resolve long-standing evolutionary questions. An example is the extinct Caribbean eulipotyphlan insectivore *Nesophontes*. Brace et al. [47] applied ancient DNA techniques to resolve the phylogenetic placement of *Nesophontes* by recovering a near-complete mitochondrial genome and 17 nuclear genes from a 750-year-old specimen. This study showed that, despite their morphological differences, *Nesophontes* formed a monophylectic clade with the extant Caribbean insectivore *Solenodon*. It further highlighted that this was an ancient divergence, as the two genera shared a common ancestor more than 40 million years ago. Other aDNA studies of an extinct island endemic include the Lava mouse *Malpaisomys insularis* [48]. Here, a more recent divergence was identified as this study revealed that *M. insularis* diverged from its morphologically incongruous closest mainland relative only 6.9 Ma.

### 3.6. Species Identification

Until the advent of ancient DNA analyses, the principle methods of identifying zooarchaeological and subfossil micromammals were via visual inspection and/or measurements taken from skeletal or dental elements e.g., [49,50]. The development of geometric morphometric methods greatly improved the sensitivity of statistical techniques for species identification but these usually incorporate some degree of error [51,52,53]. In addition, all morphological techniques are vulnerable to the confounding factors of physical damage and weak taxonomic signal. Many micromammal groups are highly species rich (e.g., Rodentia [54]) and rapid rates of diversification can result in genetic speciation without a concordant proliferation of morphological differences [55]. In addition, some species groups show high levels of phenotypic plasticity (e.g., shrews (*Sorex*) [56] and Arvicoline rodents [28,57]). When high levels of intra-specific variation are coupled with low levels of inter-specific distinction, true evolutionary relationships can be obscured due to morphological overlap.

Ancient DNA provides a crucial second line of evidence in scenarios where species cannot be easily identified on the basis of morphology alone and can also be used in isolation where morphological structures are not well preserved. However, it should be kept in mind that this approach also presents several challenges. Incorrect phylogenetic placement may occur due to low read coverage (a pervasive problem in shotgun sequenced NGS datasets with low endogenous DNA content, [30,58]), and associated incorporation of mis-called bases into the final consensus sequence. In addition, interspecific introgression of mitochondrial DNA has been commonly observed among small mammals (for example, in the rodent genus *Myodes* [59,60]) and can present significant problems in phylogenetic reconstruction [61]. Invasive sampling required for aDNA analyses are, by nature, destructive and this is particularly problematic with remains of small-bodied mammals. Molecular techniques are also limited with regards to the number of specimens that can be included in an analysis, while morphological methods can incorporate many hundreds of individuals.

Nevertheless, ancient DNA is a valuable tool in identification of species in the zooarchaeological and palaeontological record. Notably, it is particularly powerful when used to test hypotheses proposed by morphology. These studies often focus on extinct species or populations, and can provide additional data such as estimates of genetic distinction. Haring et al. [62] found that species delimitation based on genetic data from cave deposited specimens agreed with the previous morphometric analysis when examining far-eastern grey voles (Genus: *Alexandromys*). Rodrigues et al. [63] used both modern DNA and DNA extracted from museum specimens in order to test previous taxonomic hypotheses for the Egyptian weasel (*Mustela subpalmata*). This study found no evidence for genetic distinction between *M. subplamata* and the least weasel (*Mustela nivalis*) contrary to previous morphology based studies. Rodrigues et al. [63] suggested that the large size difference between these geographic populations and sexual dimorphism characteristic of *M. subplamata* are due to ecotypic rather than genetic variation. As the cost of sequencing continues to decrease, aDNA analyses become increasingly viable as a collaborative technique to identify species from archaeological sites.

### 3.7. Biogeographic Hypotheses and Island Micromammals

Micromammals are often adept dispersers and can therefore be found on many island systems [23,64]. As extinction rates on islands are higher than the mainland [65], aDNA studies play a pivotal role in our understanding of extinct or rare island micromamal evolution. Examples of studies that have used ancient DNA to examine island micromammals include: Caribbean rodents [66,67] and insectivores [47], the Easter island Pacific rat (*Rattus exulans*) [63,68,69], and the Christmas Island rat (*Rattus macleari*) [70]. A further aDNA study that examined the Orkney vole (*Microtus arvalis orcadensis*) explored colonisation dynamics, founder effects and genetic drift, all of which are associated with island populations [71].

### 3.8. Interactions with Humans

Using aDNA to explore our own human past history typically generates significant interest [72]. However, reconstructing historic human migrations can be a difficult task. Early PCR based studies were plagued by contamination from modern human samples [9] and whilst NGS techniques largely circumvent the problem, it remains relevant and validation of authenticity is required. When sample availability and permissions are also taken into account, an alternative approach to examining past human movements can be appealing. Due to their often-close interactions with humans, micromammals can make a useful proxy. Some species are domesticates (e.g., rats [73]), others have adapted to live alongside us (e.g., house mice [74]) to exploit the benefits we inadvertently provide, such as food or shelter, whilst humans have intentionally or accidentally transported and introduced other micromammals to new habitats [75]. Barnes et al. [68] analysed aDNA from the Pacific rat (*Rattus exulans*), a rodent intentionally transported by ancient Polynesians as a food item, in order to study the colonisation route of ancient humans in the Pacific.

### 3.9. Population Genetics

The use of ancient DNA in population level genetic and genomic studies can be somewhat problematic. Large numbers of individuals and/or markers are often required to fully resolve population level processes, and this is often difficult or impossible to achieve. Limiting factors in aDNA population level genetic analyses include availability/access to specimens and the variable quality of the DNA across samples. In contrast to many larger bodied species, micromammals are often found at high population densities [76], a factor that contributes to the increased likelihood of accessing larger numbers of micromammal specimens.

Hadley et al. [77] introduced a novel approach that used a combination of serial coalescent analyses with Approximate Bayesian Computation (ABC) [78] to examine past population structure, specifically a historic population bottleneck in the tuco-tucos (*Ctenomys sociabillis*). The species, a social subterranean rodent, had previously been identified as having almost no genetic variation (mitochondrial gene *cytB*) over the last 1000 years [79]. A further study [80] looked at samples from 3,000–10,000 years ago and found far higher levels of variation, suggesting a severe population decline and a bottleneck. It was to this bottleneck that Chan et al. [80] applied the ABC coalescent method. Studies such as these were amongst the first to show that aDNA could improve our estimation of the timing of events, such as population declines, allowing greater understanding of past populations and even the ability to inform practises for current populations.

### 3.10. Conservation

The use of ancient DNA data to inform conservation management and planning (termed conservation palaeogenetics; [6] or, alternately, conservation archaeogenetics; [81]) is an emerging field which holds much promise for wildlife conservation. Heterochronous aDNA datasets allow comparison of contemporary levels of genetic diversity, population size, population structure and geographical distributions with those that existed in the past [6,81,82]. This potentially allows conservation biologists to design and implement appropriate management programs based on data that is not biased by observations based only on extant populations that may be severely affected by human activity. While micromammals comprise some of the most globally abundant and widespread species (e.g., *Microtus* spp. [83]), they also contain a number of rare niche specialists (e.g., star nosed moles *Condylura cristata* [84]). To date, however, few conservation palaeogenetics studies have undertaken this focus.

Studies of Caribbean hutias [67,85] have shown that aDNA techniques can be used to analyse degraded museum and zooacheological specimens to examine living, highly endangered or rare species when sampling from wild populations is impractical. Endangered micromammals due to their small size can be particularly elusive in the wild. Museum samples can offer an alternative genetic resource to assess population structure, identify potential source populations and species boundaries to inform conservation efforts for endangered micromammal taxa [6,86,87,88]. For example, DNA extracted from museum specimens of the common hamster (*Cricetus cricetus)* allowed past levels of gene flow and population structure to be determined and this data subsequently formed the basis of population augmentation schemes in areas of the species range where it has suffered major declines [89].

### 3.11. Climate Change

Due to biological factors and their abundant presence in many archaeological and palaeontological sites, micromammals can act as excellent proxy indicators for past climate change. While early studies were constrained to use only presence-absence data (e.g., [25,26,27]), more recent research has been able to take advantage of ancient DNA evidence (e.g., [82]). Some (e.g., [90]) have combined traditional Ecological Niche Modelling with aDNA data to predict the results of future climate change. Other studies have used historic and ancient samples to investigate patterns of species continuity [35] and/or extinctions over time and fluctuating climates [91]. A further key factor in relation to climate change studies is that, prior to the large-scale, Neolithic, transition to agriculture [92], micromammals are free from the influence of anthropogenic activity. This allows the confounding factor of human-mediated effects to be removed. For example, Brace et al. [91] analysed collard lemming (*Dicrostonyx torquatus*) from Belgium over a 40,000 year time period (10–50 Kyr ago). They observed dramatic loss in genetic diversity during this period and identified a series of extinction and recolonisation events that could be related to abrupt climatic change.

## 4. Conclusions 

Molecular analysis of sub fossil material, zooacheological skeletal remains and degraded museum specimens has transformed how we explore past histories, and has the potential to continue to do so as new techniques develop. Whilst many studies continue to apply the latest techniques to charismatic species such as extinct mega fauna and humans, groups such as micromammals conversely continue to remain an understudied and untapped information resource. This is despite the large numbers of specimens available, which, due to cave environments, often exhibit superior preservation. We identify micromammals as an important future direction for the field of ancient DNA. Our study has further highlighted that, even as an understudied group, there exists a bias towards rodent species in aDNA studies of micro-mammals (see Table 1). This bias could be a result of the widespread and speciose nature of the order Rodentia, or availability of specimens and close reference sequences. Many other micromammal orders, such as eulipotyphlan insectivores, remain drastically understudied in comparison, despite their potential to resolve taxonomy and provide information on past climates and environments. The general utility of micromammal assemblages to assess the response of small mammals to past climate change, however, is becoming an increasingly important and studied area of research, the results of which can also be applied to better inform studies of present day threats to individual species and biodiversity. As ancient DNA studies continue to move away from solely species identification and taxonomy and towards answering wider evolutionary questions, the inclusion of micromammals will become increasingly important.

## Figures and Tables

**Table 1:** Published studies from 1990 to 2017 in which ancient or degraded DNA was utilised to study micromammal fauna.

Order	Genus	Species	Date	Author	Genetic Marker
Rodentia	*Dipodomys*	*panamintinus*	1990	Thomas et al. [93]	Control Region
Rodentia	*Rattus*	*exulans*	1999	Matisoo-Smith et al. [69]	Control Region
Rodentia	*Rattus*	*exulans*	1997	Matisoo-Smith et al. [94]	Control Region
Rodentia	*Rattus*	*exulans*	2004	Matisoo-Smith et al. [95]	mtDNA (~240 bp D-loop)
Rodentia	*Rattus*	*exulans*	2006	Barnes et al. [68]	Control Region
Rodentia	*Rattus*	*exulans*	2009	Matisoo-Smith & Robins [96]	Control region, Cytochrome *b*, Cytochrome Oxidase Subunit I
Rodentia	*Rattus*	*-*	2014	Robins et al. [97]	Control region, Cytochrome Oxidase Subunit I
Rodentia	*Mesomys*	*hispidus*	2003	Orlando et al. [98]	Cytochrome *b*
Rodentia	*Pappogeomys*	*alcorni*	2003	Demastes et al. [99]	Cytochrome *b*
Rodentia	*Megalomys*	*georginae, desmarestii*	2015	Brace et al. [66]	Cytochrome *b*
Rodentia	*Microtus*	*montanus*	2004	Hadly et al. [77]	Cytochrome *b*
Rodentia	*Microtus*	*longicaudus*	2009	Spaeth et al. [100]	Cytochrome *b*
Rodentia	*Microtus*	*arvalis, rossiaemeridionalis*	2012	Markova et al. [55]	Cytochrome *b*
Rodentia	*Microtus*	*gregalis*	2013	Prost et al. [101]	Cytochrome *b*
Rodentia	*Ctenomys*	*sociabilis*	2003	Hadly et al. [79]	
Rodentia	*Ctenomys*	*sociabilis*	2005	Chan et al. [80]	Cytochrome *b*
Rodentia	*Ctenomys*	*sociabilis*	2006	Chan et al. [72]	Cytochrome *b*
Rodentia	*Ctenomys*	*haigi, sociabilis*	2011	Chan & Hadley [102]	Cytochrome *b*
Rodentia	*Dinaromys*	*bogdanovi*	2007	Krystufek et al. [103]	Cytochrome *b*
Rodentia	*Glaucomys*	*volans*	2010	Kerhoulas & Arbogast [104]	Cytochrome *b*
Rodentia	*Dicrostonyx*	*torquatus*	2010	Prost et al. [82]	Control Region
Rodentia	*Dicrostonyx*	*torquatus*	2012	Brace et al. [91]	Cytochrome *b*
Rodentia	*Dicrostonyx*	*richardsoni*	2013	Fulton et al. [105]	Cytochrome *b*
Rodentia	*Dicrostonyx*	*torquatus*	2013	Prost et al. [101]	Cytochrome *b*
Rodentia	*Dicrostonyx*	*groenlandicus, richardsoni, torquatus*	2016	Palkopoulou et al. [106]	Cytochrome *b*
Rodentia	*Peromyscus*	*polionotus*	2012	Kalkvik et al. [107]	Cytochrome *b*
Rodentia	*Plagiodontia*	*aedium*	2012	Brace et al. [85]	Cytochrome *b*
Rodentia	*Ototylomus*	*phyllotis*	2014	Gutierrez-Garcia et al. [39]	Cytochrome *b*
Rodentia	*Alexandromys*	*fortis, indet, maximowiczii, oeconomus*	2015	Haring et al. [62]	Control Region
Rodentia	*Pennatomys*	*nivalis, luciae*	2015	Brace et al. [66]	Cytochrome *b*
Rodentia	*Arvicola*	*amphibius*	2016	Brace et al. [108]	Control Region
Rodentia	*Malpaisomys, Mus, Murinae*	*-*	2012	Pages et al. [48]	Cytochrome b, Interphotoreceptor Retinoid Binding Protein (IRBP)
Rodentia	*Mus, Meriones, Apodemus, Lemniscomys, Rattus, Gerbillus, Cricetulus, Microtus, Eliomys*	*-*	2016	Guimaraes et al. [35]	Single Nucleotide Polymorphisms (SNPs)
Rodentia	-	-	2002	Kuch et al. [109]	12S & 16S rDNA, Cytochrome *b*
Diprotodontia	*Bettongia*	penicillata	2015	Pacioni et al. [110]	Control Region, Microsatellites
Eulipotyphla	*Sorex*	*araneus, tundrensis*	2013	Prost et al. [111]	Cytochrome *b*
Eulipotyphla	*Erinaceus*	*europaeus*	2012	Fraser et al. [112]	Cytochrome *b*
Eulipotyphla	*Nesophontes*	*paramicrus*	2016	Brace et al. [47]	Mitochondrial genome, ADORA3, ADRA2B, ADRB2, APOB, APP, ATP7A, BCHE, BDNF, BMI1, BMP4, BRCA1, CREM, EDG1, GHR, PLCB4, RAG1, RAG2, RHO, TTN, TYR, VWF
-	-	-	2005	Avenant et al. [40]	Cytochrome *b*
-	-	-	2005	Poulakakis et al. [45]	Cytochrome *b*
-	-	-	2016	Guimaraes et al. [46]	MtDNA
-	-	-	2008	Tougard & Renvoise [113]	
